# Spatio-Temporal Patterns of Key Exploited Marine Species in the Northwestern Mediterranean Sea

**DOI:** 10.1371/journal.pone.0037907

**Published:** 2012-05-24

**Authors:** Marie Morfin, Jean-Marc Fromentin, Angélique Jadaud, Nicolas Bez

**Affiliations:** 1 UMR 212 EME, IFREMER (Institut Français de Recherche pour l'Exploitation de la mer), Sète, France; 2 UMR 212 EME, IRD (Institut de Recherche pour le Développement), Sète, France; University of Western Ontario, Canada

## Abstract

This study analyzes the temporal variability/stability of the spatial distributions of key exploited species in the Gulf of Lions (Northwestern Mediterranean Sea). To do so, we analyzed data from the MEDITS bottom-trawl scientific surveys from 1994 to 2010 at 66 fixed stations and selected 12 key exploited species. We proposed a geostatistical approach to handle zero-inflated and non-stationary distributions and to test for the temporal stability of the spatial structures. Empirical Orthogonal Functions and other descriptors were then applied to investigate the temporal persistence and the characteristics of the spatial patterns. The spatial structure of the distribution (i.e. the pattern of spatial autocorrelation) of the 12 key species studied remained highly stable over the time period sampled. The spatial distributions of all species obtained through kriging also appeared to be stable over time, while each species displayed a specific spatial distribution. Furthermore, adults were generally more densely concentrated than juveniles and occupied areas included in the distribution of juveniles. Despite the strong persistence of spatial distributions, we also observed that the area occupied by each species was correlated to its abundance: the more abundant the species, the larger the occupation area. Such a result tends to support MacCall's basin theory, according to which density-dependence responses would drive the expansion of those 12 key species in the Gulf of Lions. Further analyses showed that these species never saturated their habitats, suggesting that they are below their carrying capacity; an assumption in agreement with the overexploitation of several of these species. Finally, the stability of their spatial distributions over time and their potential ability to diffuse outside their main habitats give support to Marine Protected Areas as a potential pertinent management tool.

## Introduction

The Mediterranean Sea is a marine biodiversity hotspot [1]–[3] that is subject to growing anthropogenic pressures, such as fisheries, eutrophication [4] and climate change [5], [6]. This study focuses on the Gulf of Lions, the northwestern area extending along the French coast, which is particularly exposed to anthropogenic pressures due to its high population density and fishing activity. Despite the direct adverse effects of bottom trawling on the seabed and bentho-demersal biotopes [7], this region still hosts a high diversity of species [8]. However, several exploited species suffer from (growth) overexploitation [9].

Beside traditional management measures (e.g. minimum net mesh size, time at sea, number of fishing boats [10]), Marine Protected Areas (MPA) are a tool used for conservation purposes (i.e. to protect marine natural resources from anthropogenic impact) and/or for maximizing ecological services (as expected under a sustainable exploitation of those resources) [11], [12]. In many cases, MPAs are designed without adequate information regarding the functioning of the local ecosystem, thus failing to meet the objectives for which they were implemented in the first place [13]. Modeling both fishing effort and spatial distributions of the exploited species can help stakeholders and managers in the decision of where to place the MPAs [14]. This study focuses on this second aspect and aims to describe and quantify the spatial dynamics of benthic-demersal marine resources in the Gulf of Lions (Northwestern Mediterranean Sea), using the 17 years of scientific time-series surveys carried out in this region. Previous investigations examined the spatio-temporal patterns of fish assemblages of demersal species, which are primarily distributed along a depth gradient (from the coastal zone to the upper slope) and then along a longitudinal gradient [15]–[17]. From a temporal viewpoint, the spatial patterns of these assemblages of multiple fish species were relatively stable from 1983 to 1992. However, no single species analysis has been performed so far. Our aim is thus to rigorously quantify the temporal stability/variability of the spatial distributions of key exploited species since 1994, which is of interest from a MPA perspective.

To do so, we proposed a geostatistical approach to handle zero-inflated and non-stationary distributions and to test for the temporal stability of the spatial structures of each species. Empirical Orthogonal Functions (EOF) and other indicators were then applied to investigate the temporal persistence and characteristics of the spatial patterns.

## Materials and Methods

### Survey design

**Figure 1:**
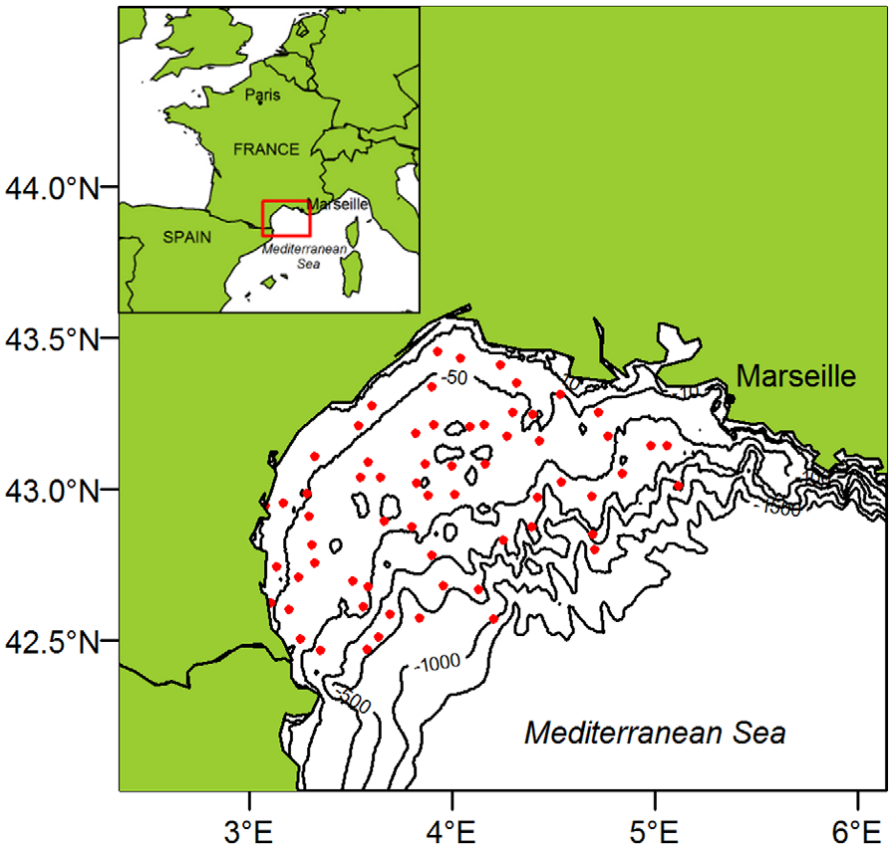
Study area and sampling sites. Map of the Gulf of Lions and the 66 sampling sites (identified by a red point) during the whole MEDITS survey (1994–2010). The positions of the sites were fixed by a stratified sampling scheme.

Data was collected within the “International Bottom Trawl Survey in the Mediterranean Sea” (MEDITS) project [18] conducted every year since 1994 in May and June. In the Gulf of Lions, 66 stations were fixed according to a stratified random sampling based on five depth strata (10–50 meters, 50–100 m, 100–200 m, 200–500 m, 500–800 m) divided in East-West sub-strata by 4°E longitude (Fig. 1). Hauls were performed in daylight following a standardized protocol. The hauls lasted 30 minutes for shelf stations (10 m–200 m) and 60 minutes on the upper slope (>200 m, to compensate for a lower catchability on irregular grounds). Geo-referenced position, speed and distance covered by the trawls were systematically recorded. The experimental net (GOC 73) used for sampling had a 20 mm-diamond stretched mesh size at the cod-end [19]. An underwater Scanmar system was used to control the trawl geometry and take off the analysis tows not properly sampled. Horizontal and vertical openings of the gear were about 18 m and 2 m, respectively. The catch contents were sorted, counted and weighted by species. For the 35 species of reference listed [18], the length and sexual maturity were also recorded. The survey finally provided the density of individuals for each species, obtained by dividing the counts observed by the trawled surface. This is an indicator of the local abundance relative to the trawl catchability, which is assumed to be constant.

### Key species

Since the onset of the MEDITS survey, 300 different species have been identified in the Gulf of Lions, but many are low in abundance or even rare [20]. Describing the spatial distributions and evaluating temporal variability with statistical tools could hardly be done for rare species. We therefore decided to focus on several species according to four criteria: (i) catchability, i.e. keeping only the species that are properly sampled by the MEDITS gear; (ii) level of abundance, i.e. keeping only the species that are relatively abundant in the Gulf of Lions (106 species are present in more than 5% of the hauls); (iii) commercial importance, i.e. focusing on exploited species; and (iv) taxonomic/trophic positions, i.e. including different trophic levels and/or taxonomic groups, such as fish, cephalopods, decapods and elasmobranchs. This selection led us to retain the following 15 species: European hake (*Merluccius merluccius*), angler (*Lophius piscatorius*) and black-bellied angler (*Lophius budegassa*), Atlantic horse mackerel (*Trachurus trachurus*), Mediterranean horse mackerel (Trachurus mediterraneus), grey gurnard (*Eutrigla gurnardus*), red gurnard (*Aspitrigla cuculus*), Norway lobster (*Nephrops norvegicus*), red mullet (*Mullus barbatus*), horned octopus (*Eledone cirrhosa*), small-spotted catshark (*Scyliorhinus canicula*), elegant cuttlefish (*Sepia elegans*) and three squid species belonging to the Teuthoidea Order (*Illex coindetti*, *Todarodes sagittatus* and *Todaropsis eblanae*). Because of identification problems for juvenile anglers and squids, we had to put the two angler species into one group and do the same for the three squid species. We decided to include these 5 species because they are important targets for fisheries. Finally, we retained 12 species-like categories (later called key species).

### Statistical analysis

Our goal was to describe the spatial distributions of the selected key species in the Gulf of Lions and to evaluate their temporal variability. In order to analyze the whole 204 distributions sampled (17 years and 12 species), we chose a spatial statistical method that is valid for all the species. Firstly, we analyzed the spatial structures of the annual observations of the 12 selected key species. Indeed, many animals have a tendency to aggregate, for social reasons or because they depend on the same external factors, which are also spatially structured (preys, physical variables, etc). The spatial structure is thus the value of the spatial autocorrelation at different lags (distance intervals). This led us to use geostatistics, a common technique to analyze the spatial patterns from in-situ data [21]. In this context, the spatial structure is modeled by the variogram, which specifically measures the variance of the difference between observations at two locations at any lag across the realization of the field. The spatial structure (in other words, the spatial autocorrelation) is thus only one characteristic of the spatial distribution of species. Indeed, a population may present – from one year to another – peaks of densities in different parts of the field (i.e. displaying different spatial distributions), while keeping the same underlying structure. The second step was to assess the temporal stability of the annual spatial structures. In other words, we tested if the 17 annual empirical variograms of each species were similar to each other. The third step of the study was to map the spatial distributions of the different (groups of) species by interpolating each set of annual observations over the study area, using the variogram (i.e. by kriging). Finally, we described the spatio-temporal patterns of these maps using EOF method and other indicators. All statistical analyses were conducted using the R software [22] and the geostatistical analyses were performed with the package RGeoS (http://cg.ensmp.fr/rgeos).

#### Non-stationary distributions

Empirical variograms from the raw data of the 12 key species did not lead to satisfactory results. The variograms were either flat or highly variable from one class of distance to another. It appears that some underlying stationary assumptions were violated due to two reasons: the presence of large areas with zero values and a proportional effect.

The histograms of species densities were highly skewed (coefficients of variations ranging from 0.7 to 7.1), characterized by large proportions of zero (or low) values and by very low proportions of extremely large values. Skewness of distributions is symptomatic of a proportional effect [23], i.e. situations where the standard deviation of local densities is proportional to the corresponding average density [24]–[26]. This was the case for the 12 key species for which the standard deviations of their densities per stratum were proportional to the averages per stratum. To correct for such a pattern, we applied the Box-Cox transformation to the densities of the 12 key species [27], [28]. For the 12 key species and 17 years, the optimal transformation was the logarithm or very close shapes. For homogeneity purpose, densities (denoted Y) were thus systematically log-transformed: *Z* = log(*Y*+*c*), where c is a positive constant and log is the natural logarithm. We set c to the smallest observable density to generate the smallest impact on the form of the variogram:
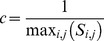
where S_i,j_ is the trawled surface at station i and of year j. The results and interpretations are done on the logarithm scale, and the term ‘log-density’ refers to the variable Z as described above.

For some species, this transformation did not totally resolve the issue of stationarity because of the presence of many locations with zero values. The large proportion of zero values does not contradict the stationarity assumption, unless they are concentrated in the same area, which was the case in this study. Zero values in the observations may appear in two contexts: (i) the species was actually absent at the sampled site, thus a zero value will be always sampled in the neighborhood, or (ii) the trawl did not catch the individuals because of their scarcity, mobility and aggregative behavior, so it will appear in areas with small densities values. The stationarity assumption is violated only in the former case, thus the sub-areas should be removed from the analysis, but in practice there is no objective criterion to distinguish the underlying process. Thus we only excluded sub-areas where no individual has been caught during the 17 years (1994–2010). To get homogeneous areas, we only considered contiguous sites with zero values according to the bathymetry. To do this, we removed all the sites of depths smaller than 70 m, 310 m and 350 m, for the Norway lobster, the cuttlefish and the red mullet, respectively (we kept the full area for the other 9 key species).

For comparison purposes, the analysis was also performed on presence/absence data. Binomial presence/absence data is relevant in the case of many zero values and it also avoids a proportional effect. Maps produced with such variables are spatial predictions of the probability of the presence of at least one individual. Furthermore, it enabled us to calculate presence/absence areas, which are an interesting indicator of spatial distribution (see below).

#### Analysis of the spatial structure and mapping

We calculated the empirical variogram on the transformed variable Z to investigate the spatial structure (i.e. the spatial autocorrelation, see above) of each species for each year. The distance lag was set to 9 km, which corresponds to the minimum distance including at least 5 sites. The empirical variogram is associated with a sampling variance, which quantifies the uncertainty to assess the underlying true variogram, owing to the limited number of available samples (62 on average). For this reason, we examined if the annual empirical variograms of a given species were significantly different from year-to-year, in comparison with the sampling variance associated with the mean variogram. To do so, we first estimated the mean variogram of each species, by averaging the 17 annual empirical variograms at each distance lag. Then, the mean empirical variogram of each species was modeled using curves with known properties, as is common practice [29]. Lastly, we simulated Gaussian random fields according to the mean modeled variogram, using the turning-bands method [30]: each field is generated by dividing the area in many lines on which are simulated values according to a certain covariance, derived from the modeled variogram. For each simulated field, we retained the values at the 66 locations of the survey and then calculated the corresponding empirical variogram. Note that the simulated empirical variogram would be equal to the modeled version if it would be calculated on a sufficiently large number of points. We simulated 500 Gaussian random fields, so that we got 500 simulated empirical variograms that were then used to determine the confidence interval around the mean modeled variogram. Finally, we compared the variability of the 17 observed variograms against the range of variability obtained by the 500 simulations. If some annual variograms were outside the confidence interval of 95% defined by the simulated variograms, we concluded there were significant year-to-year differences while if the 17 empirical variograms remained within the interval, we assumed that these 17 variograms did not differ significantly from the mean variogram (variations are within what it is expected at random). Such a test allowed us to: (i) investigate the temporal variability/stability of the spatial structure of each species and (ii) perform the kriging with a mean variogram (if the spatial structure is stable) that is more robust (less noisy) than the annual variograms. Annual maps of log-density were then produced by kriging annual observations on a 1 km square-mesh grid over the study area using the annual or mean variogram depending on the results of the above test.

#### Juveniles versus adults analysis

We also produced distribution maps of juvenile and adult stages over the 17 years. A year-to-year analysis was not possible because the densities of adults for many species were too low. Even so, such an analysis could be not done for squids, anglers, cuttlefish and red gurnard because: (i) squids and anglers included two to three species (see above) that display different length-at-maturity, (ii) the length and the maturity stages were not estimated for cuttlefishes and (iii) adult abundance was too low over the whole time period for red gurnard. For the nine remaining species, we kriged the means of the log-densities Z (or presence P) at each station over the 17 years. Juveniles and adults were discriminated using the length at 50% of maturity (L50) from Sardà [31] for Norway lobster, from Quéro and Vayne [32] for red mullet and calculated from MEDITS dataset for the other species.

#### Empirical Orthogonal Functions

The Empirical Orthogonal Functions (EOF) method was first applied to quantify the temporal stability of the spatial patterns in a more objective manner than simple visual inspection, as it is usual [33]. EOF analysis is equivalent to a Principal Component Analysis applied to spatio-temporal data. In our case, the dataset for each species included the log-densities of the annual kriging maps, with time (years) as descriptors and space (pixels) as objects. The EOFs were then performed on the correlation matrix among the objects to compare the annual distributions independently of their level of abundance. The first EOF is the linear combination of the years which maximizes the variance of the spatial distributions and its percentage of variance is (when the contributions of the descriptors are all positive or all negative) an indicator of the temporal stability/variability of the spatial distributions, i.e. the greater the percentage of variance, the higher the temporal stability of the spatial distributions [34].

#### Descriptors of spatial distributions

Several indices have been used to characterize the spatial distributions. Centre of gravity and inertia are two simple indices able to detect spatial shifts [35]. In addition to this, we investigated MacCall's “basin hypothesis”. According to the Ideal Free Distribution (IFD) theory, individuals ultimately settle into the areas that allow them to achieve equal and maximum fitness [36]. The fitness of an individual increases with habitat suitability, which depends on prey availability, predation and abiotic habitat characteristics. As the local abundance increases, individuals spread into less suitable habitats because of intra-specific competition. MacCall [37] applied the IFD model to a pelagic fish population and demonstrated how geographical range expands/contracts as abundance increases/decreases. The theory assumes that the link between habitat suitability and abundance is positive, but it can be linear or non linear [38], so that working on the logarithm scale is appropriate, as it is a monotonically increasing function. To investigate MacCall's basin theory, we analyzed the relationship between the presence area and the log-abundance. The presence area (PA) was calculated from the maps of presence/absence as the number of pixels displaying a value >0.5 (i.e. a probability of presence exceeding 50%) divided by the total number of pixels. Therefore, PA is given as a percentage (from 0% to 100%) and may be seen as the spatial range of each species for each year relative to the whole studied area. The estimation of the log-abundance (Z_T_) was estimated from the kriged data 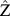 as follow:
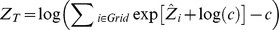
Back-transforming log-densities before summing them over the area provide a more adequate estimation of the log-abundance, than directly summing log-density values. Indeed, the logarithm of a sum of terms is different from the sum of logarithm of the terms. Note that we could have also calculated abundance using adequate back-transformation formulation for kriged data [28], provided that they are log-normally distributed. However, such back-transformation is very sensitive to the shape of data distribution and can lead to highly biased estimates when the transformed data are not perfectly normally distributed, which is the case when data are zero-inflated.

## Results

### Spatial structure

**Figure 2:**
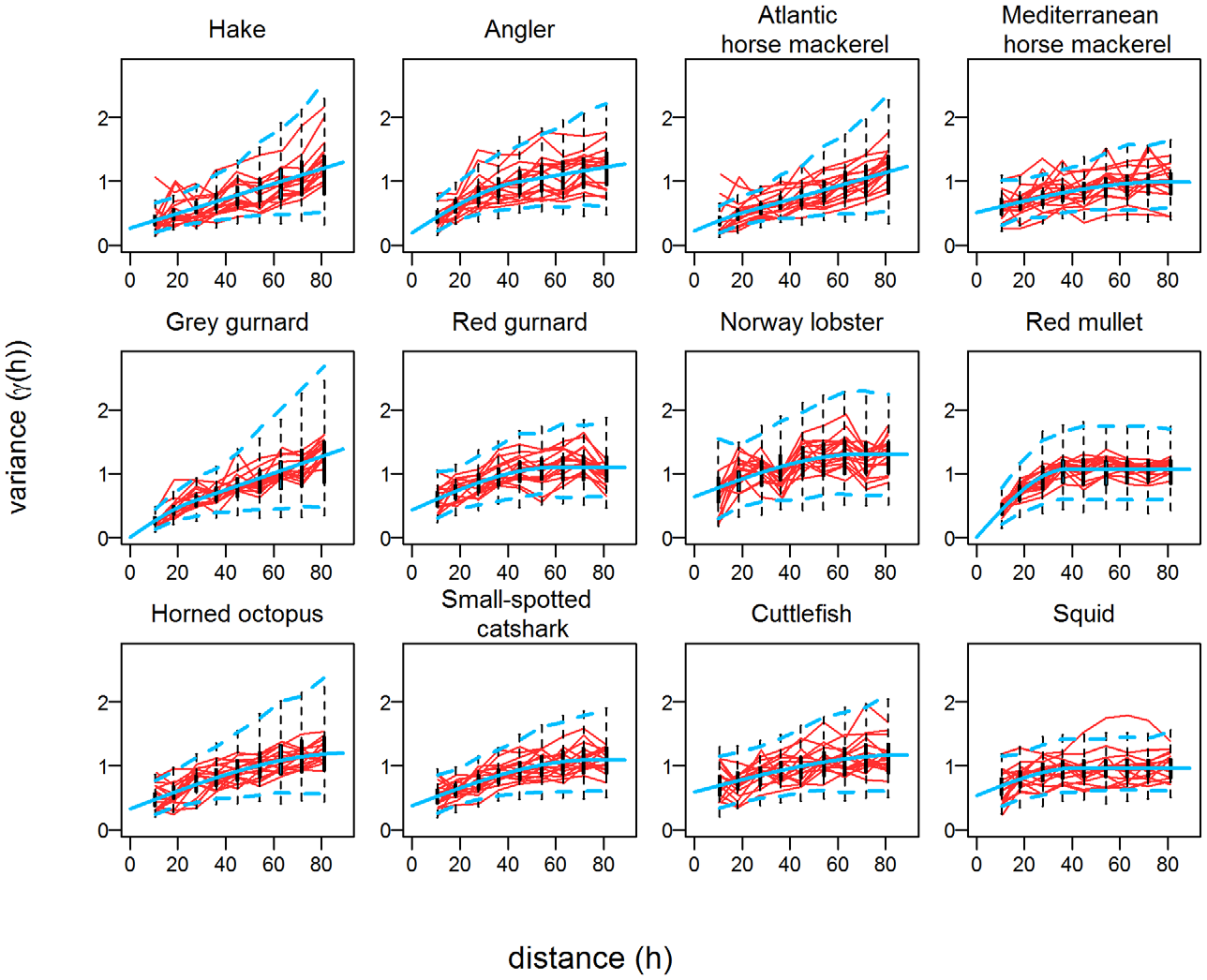
Variograms analysis. Annual empirical variograms from 1994 to 2010 (red) and model fitted to mean (over the 17 years) empirical variogram (blue). Intervals of variability (blue dashed line) at 95% were calculated from 500 Gaussian fields simulated according to the model of the mean variogram at the 66 survey stations. x-axis: distances (in km), y-axis: variance of the difference between field values.

Annual empirical variograms on log-density values depicted clear spatial structures (Fig. 2). For all the species, except hake for which variograms increased linearly with distance, the variograms can be modeled by a spherical function that stabilized around a sill or had an additional linear structure starting from a distance range. For each species, the shapes of the annual variograms were very similar, with some years apparently less structured than others. The simulation procedure indicated that the 17 annual variograms were all included in the 95% confidence interval for all the species, except one for cuttlefish (Fig. 2). Consequently, we concluded that there was no significant difference between the 17 annual variograms of a given species. In other words, the spatial structure, as estimated from the variogram, appeared to be stable from year-to-year for the 12 key species. Therefore, mean annual variograms were systematically used to krige the annual log-densities.

#### Annual maps of density

**Figure 3:**
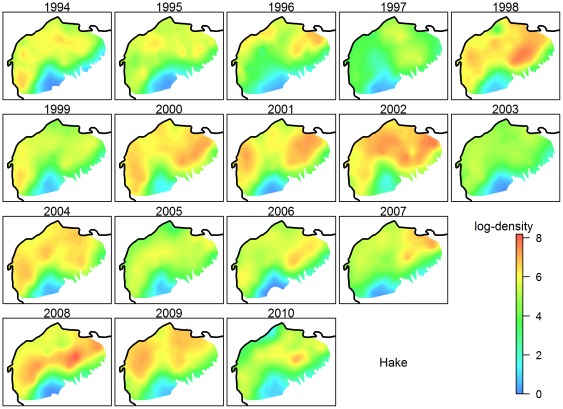
Annual maps of hake. Kriged annual maps of European hake (*Merluccius merluccius*) log-density (Z) from 1994 to 2010.

**Figure 4:**
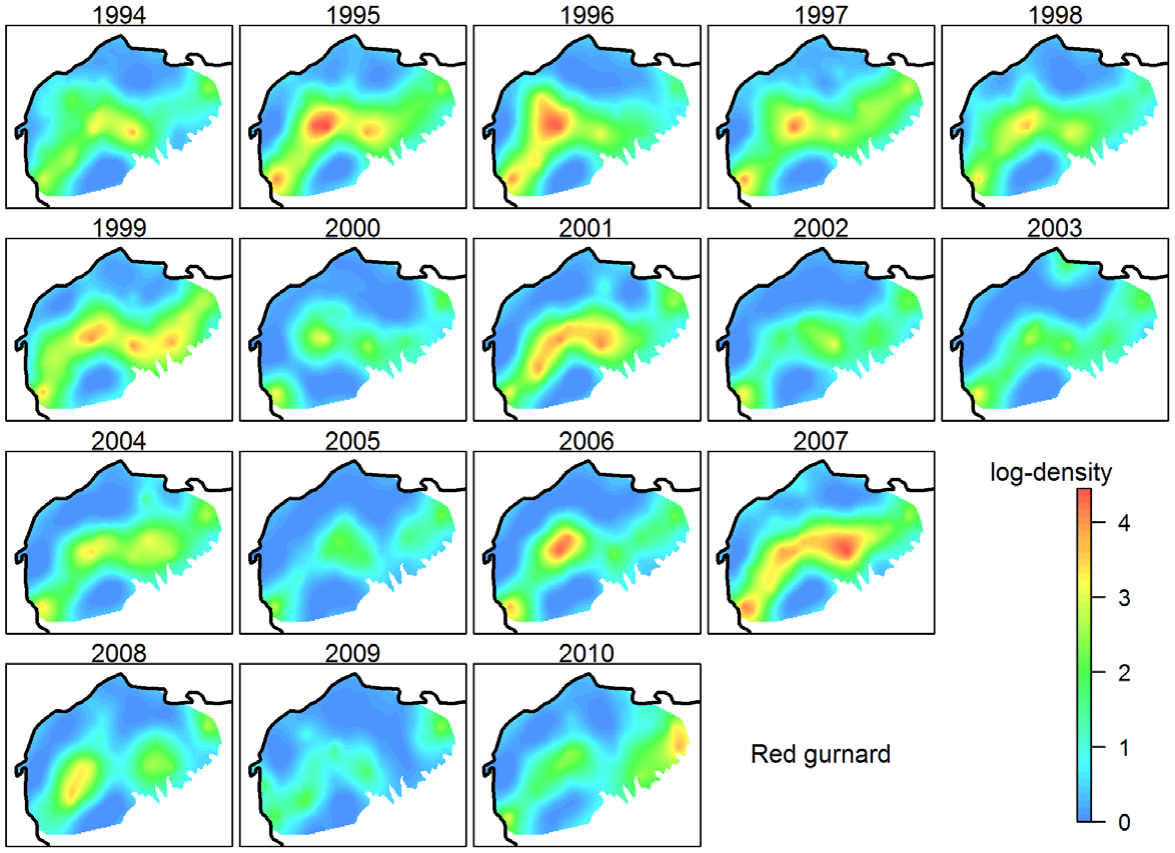
Annual maps of red gurnard. Kriged annual maps of red gurnard (*Aspitrigla cuculus*) log-density (Z) from 1994 to 2010.

Figures 3 and 4 show distributions of hake and red gurnard, respectively, as examples (see Figs. S1,S2,S3,S4,S5,S6,S7,S8,S9,S10 for the other species). At first glance, the spatial distributions of log-densities of any given species were surprisingly similar from year-to-year. Hake was present homogeneously in the whole study area, except in the southwestern corner along the continental slope (Fig. 3). Patches of higher log-density appeared some years, such as in 1998, 2000–2002 and 2008, mostly in the same eastern areas of the continental shelf. In contrast to hake, red gurnard only occupied a fraction of the study area (Fig. 4). This species is absent onshore and offshore in the same southwestern corner as hake. Spots of high log-density also appeared (in 1995, 1996, 2001, 2006 and 2007) along the same central arch of the shelf. This high temporal stability of the spatial distributions was found for all the species, except for squid, which were distributed in different locations (possibly because this group includes three different species). Cuttlefish and Mediterranean and Atlantic horse mackerels were exclusively inshore (<200 m); angler, grey gurnard, catshark and horned octopus were more offshore (50 m–500 m, see Fig. S1,S2,S3,S4,S5,S6,S7,S8,S9,S10). Norway lobster was found all along the shelf break, but also at low densities on the eastern center of the shelf, since 1996. Red mullet was found in shallow waters (<50 m) and on the shelf between depths of 100 m and 200 m and displayed a patchy distribution. Small-spotted catshark was also distributed heterogeneously, in two patches on both sides of the shelf. In general, the western side of the slope of the Gulf exhibits extremely low log-densities, except for Norway lobster. Inside their presence area, many species were not fully homogeneously distributed and spots of higher log-density appeared at various locations some years. If the spatial distributions of a given species were relatively stable through time, presence areas seemed to extend some years. Furthermore, they appeared dissimilar from one species to another and the spatial dynamics were also different: years of high/low log-abundance were not synchronous among the 12 key species.

#### Juvenile versus adult analysis

**Figure 5:**
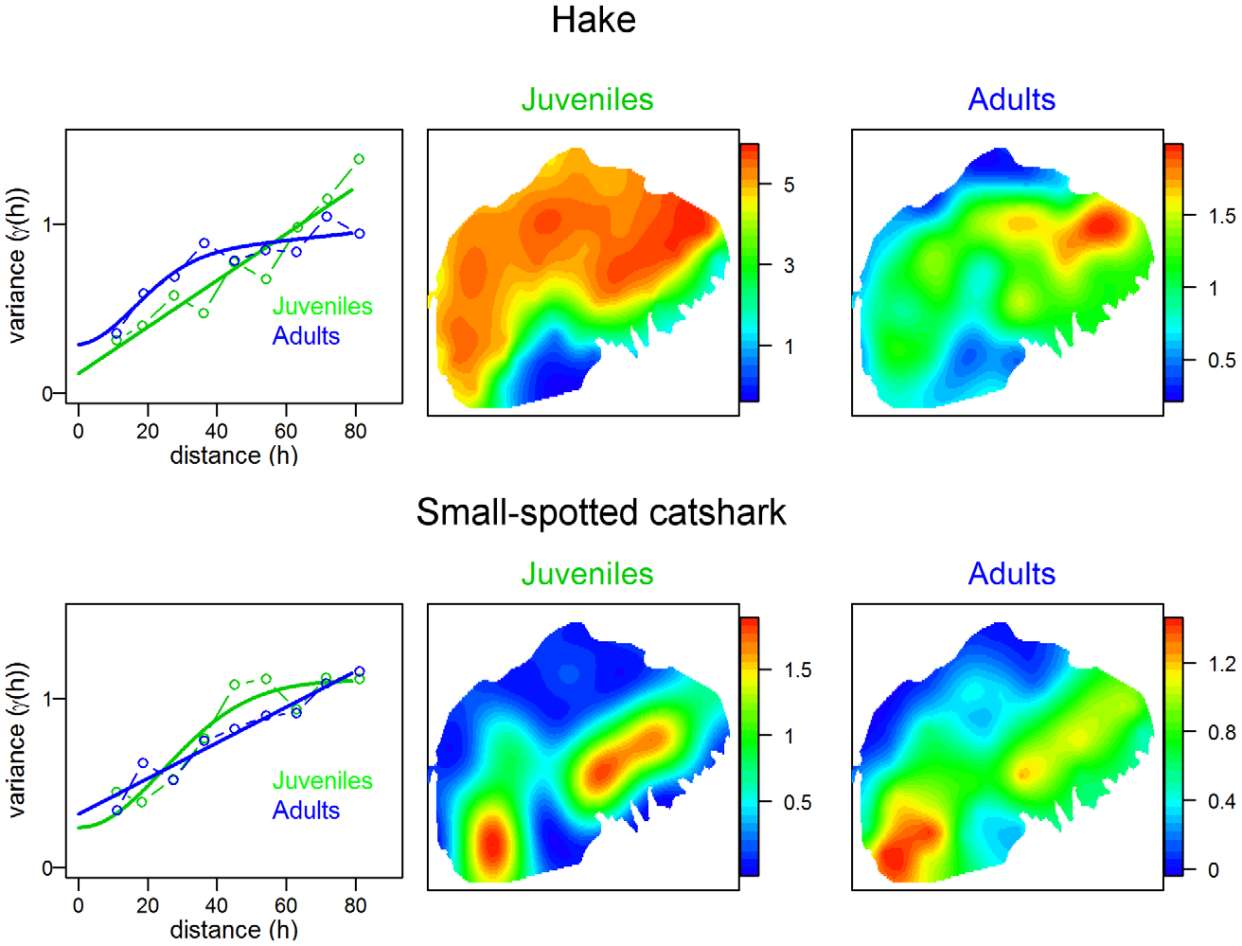
Juvenile *versus* adult stages comparison. Average maps (1994–2010) of the log-density (Z) of juveniles and adults of the European hake (*Merluccius merluccius*) and the small-spotted catshark (*Scyliorhinus canicula*). In the first column the corresponding variograms are displayed.

For nine retained species we mapped the average log-density of adult and juvenile stages over the survey period. Figure 5 displays variograms and spatial distributions of hake and small-spotted catshark, which represent both typical patterns encountered among species (see Fig. S11 for the others). Hake adults were more concentrated than juveniles and in deeper western waters, but were included within the distribution range of the juveniles. This was also the case for Atlantic horse mackerel and horned octopus. The spatial distributions of both maturity stages were nearly identical for catshark, Norway lobster, Mediterranean horse mackerel, grey gurnard and red mullet.

#### Empirical Orthogonal Functions analysis

**Figure 6:**
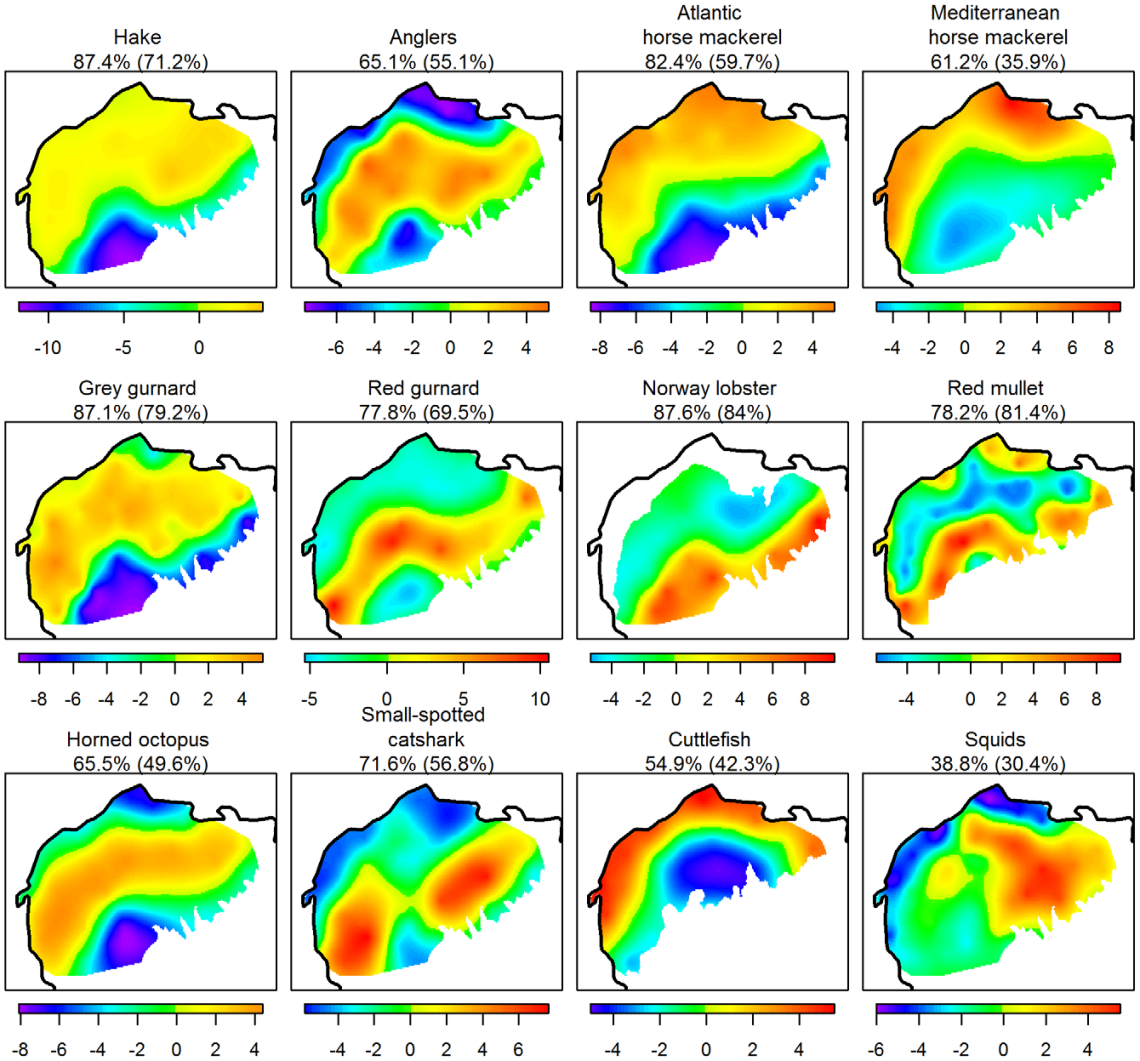
Empirical Orthogonal Function analysis. Projections on the first Empirical Orthogonal Function performed on kriged maps of log-density of each species (or group of species) from 1994 to 2010. The percentage of variance explained by this first axis is a proxy of the stability of the spatial distributions (see text). In parentheses are given, for comparison purposes, the percentages obtained from EOF applied on raw data.

EOFs were applied on the annual maps of each species to assess the temporal persistence of the spatial patterns (see Statistical analysis section). The percentage of variance explained by the first axis of the EOF was in general high (71% on average), but varied among species, i.e. from 39% (for squid) to 88% (for Norway lobster, Fig. 6). The correlations between the annual distributions and the first EOF axis (Fig. S12) showed that contributions of the descriptors (i.e. the years) to this axis were relatively balanced and all positive, meaning that the percentage of explained variance is a good index of stability of the annual distributions through the whole period. Figure 6 displays the projections on the first EOF axis for each species, which thus depict the most stable spatial pattern. We can clearly recognize the persistent areas of high log-density (in red), areas of absence (in blue) and areas more variable between years (in green and orange), as observed on annual maps. The EOF clearly shows that the spatial distributions of the great majority of the 12 key species were highly stable from year-to-year, but differed among species (which is consistent with the above findings). EOFs were performed on kriged values which are generally smoother than raw data (i.e. observation at each sampling site), so that it may affects the percentage of variance given by the first EOF axis. In other words, the kriging process could have artificially increased the degree of stability. To investigate this potential bias, we also applied the EOFs directly on the raw data. Percentages of variance obtained were also high (Fig. 6), though globally lower (60% in average) except for the red mullet (81%, while 78% for kriged data). Species were ranked in the same order, expect for both mackerels. Overall, results did not show any systematic bias when the EOFs were computed on kriged data.

#### Descriptors of spatial distributions

**Figure 7:**
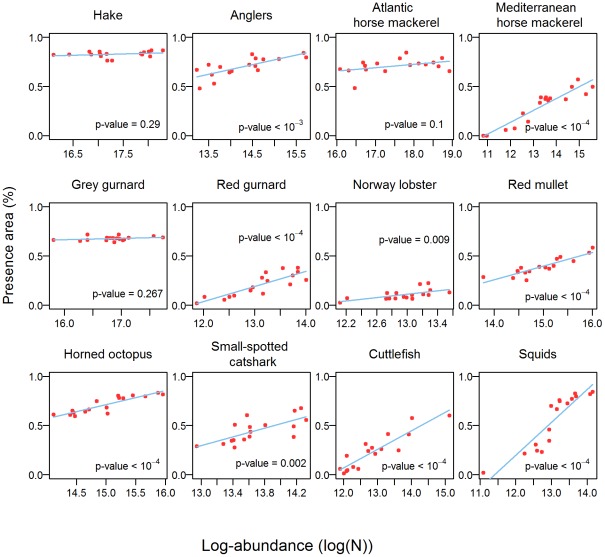
Presence areas. Linear regressions between the presence area (given as a percentage of the whole study area) and log-abundance, for each key species.

Centers of gravity and inertia calculated annually were very stable for each species compared to shifts between species distributions (Fig. S13). Moreover, no directional trend was detected between 1994 and 2010. On the contrary, the presence area (PA), were highly variable for many species (Fig. 7); squids being the most extreme case, with percentages of PA varying from 0.02% to 97%. Globally, many species were rather ubiquitous on the scale of the Gulf of Lions, except Norway lobster, red gurnard and Mediterranean horse mackerel (whose maximum occupancies were 35%, 40%, and 60%, respectively). The presence areas also varied between species, from averages of 12% (Norway lobster) to 96% (hake).

**Figure 8:**
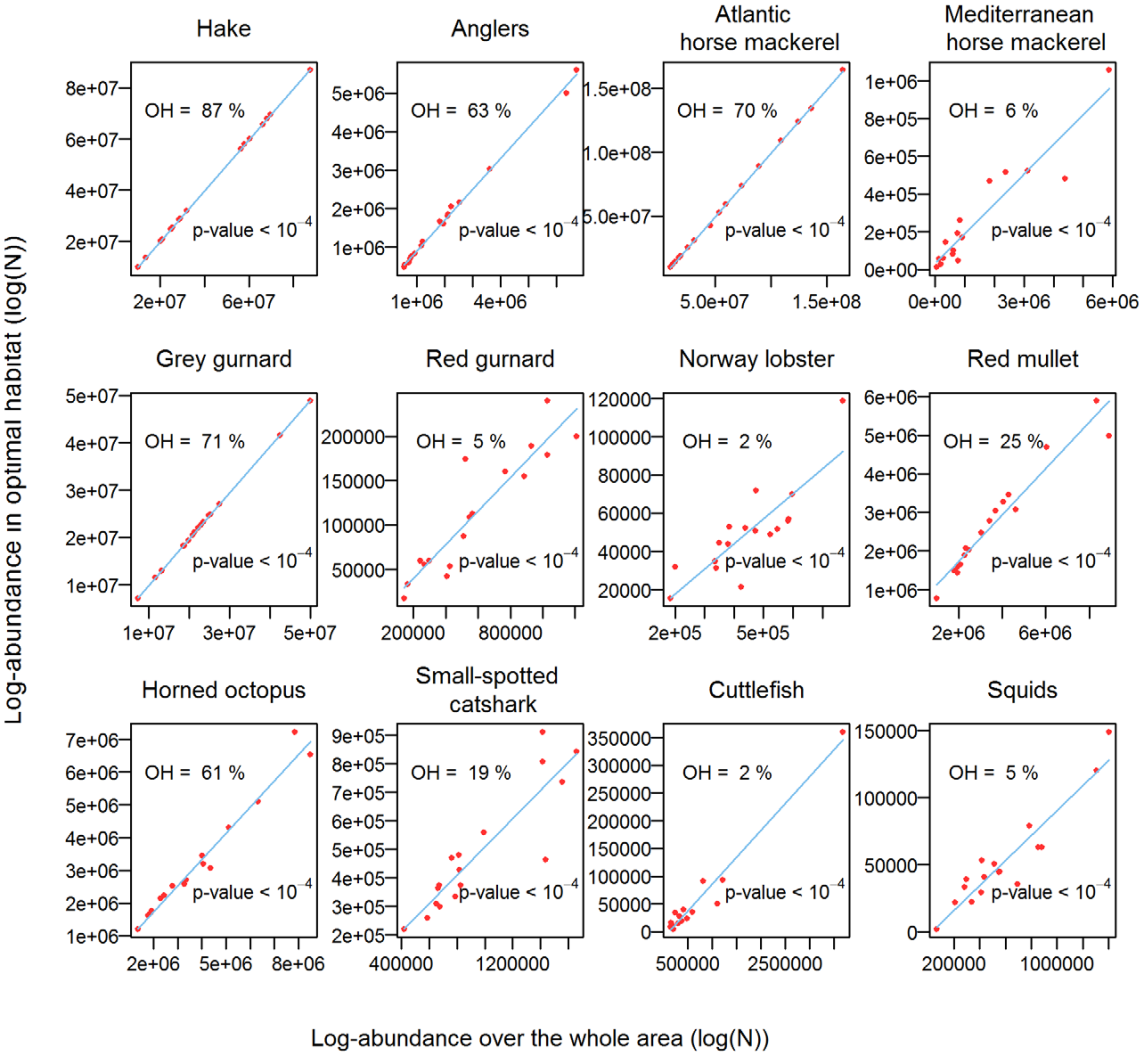
Log-abundance in optimal habitats. Linear regressions between the log-abundance inside the optimal habitat and log-abundance over the whole area, for each key species. OH is a percentage that reflects the surface covered by the optimal habitat divided the surface of the whole study area (26 716 km^2^).

For all 12 key species, the relationships between PA and Z_T_ were linear and positive (Fig. 7). In most cases, Pearson's correlations were significant and high (p-values<5%), except for hake, grey gurnard and Atlantic horse mackerel. However, these 3 species also displayed the largest PA, which makes more difficult to detect any expansion/contraction. To complete the above analysis, we also calculated the log-abundance in the sub-area where the species persisted the most over time (defined as the pixels for which the species has been detected in at least 12 of the 17 years, i.e. 75% of the time). These sub-areas were supposed to be the most optimal habitats. The size of these sub-regions varied among species, from 2% (Norway lobster) to 87% (hake) of their presence area (Fig. 8). Log-abundance in those optimal habitats and log-abundance over the whole area displayed positive linear relationships for all the species (Fig. 8). In other words, when the log-abundance of species increased over the whole study area, it also increased in their optimal habitats. Note that for 3 species (i.e. hake, grey gurnard and Atlantic horse mackerel), the sizes of the optimal habitats were large and close to these of the presence areas (>70%). However, for species like Norway lobster and cuttlefish, which main habitat only covered 2% of their presence area, the relationships were also significantly positive linear.

## Discussion

This study aimed at describing the spatio-temporal characteristics (i.e. the spatial structures and the distribution maps) of 12 key benthic/demersal species in the Gulf of Lions to evaluate the temporal stability/variability of the spatial patterns. We produced annual maps of log-densities for each key species by kriging bottom trawl survey data collected annually from 1994 to 2010, while the spatial structures were taken into account using variograms. Geostatistics [21], [28] allowed us to characterize the spatial dependence of observations and to predict a variable at any unobserved location of the study area, accounting for this dependence. This has shown to be useful when dealing with fishery survey data [39], [40]. Nevertheless, such data often has undesirable features (many zero and/or extreme values), which make using geostatistical tools challenging [41]. Underlying assumptions of geostatistics were unfulfilled because of too high proportions of many zero values and some proportional effects. To handle the “zero” issue, we removed, for three species, homogeneous areas where the species were always absent during the 17 successive years. In addition, the annual densities were normalized by a natural logarithmic transformation that removed the proportional effect. Resulting variograms were much more structured. For the same reason, Maynou et al. [42] obtained variograms for Norway lobster densities by re-scaling the local variance with the local mean [43], which were even more structured than the variograms obtained on log-transformed data. However, this method is sensitive to the (subjective) choice of sub-areas to calculate local means. We will show below that our results are robust to the data transformation.

One of the objectives of this study was to test the stability/variability of the spatial structures over time. To do so, we developed an original approach based on geostatistical non-conditional simulations of spatial Gaussian processes defined by the mean variograms obtained during the data-analysis phase. By calculating empirical variograms at the 66 sampling sites, we simulated the uncertainty inherent to stochastic models that comes from the fact that we used an incomplete census of the study area. It is interesting to note that the Box-Cox transformation of the data insured a consistency of the entire simulation exercise. Simulations are theoretically founded for Gaussian processes, which are the only situations where the knowledge of a variogram amounts to the full characterization of the model. The log-transformed densities were reasonably Gaussian, so that comparing the variograms obtained from log-transformed data and the variograms obtained by (non-conditional) simulations was relevant. The results clearly showed that the variograms were all included in the 95% confidence intervals (except for squids in 2000), revealing stable spatial autocorrelation patterns for each species. The presence of spatial structure had already been detected in other regions, for Norway lobster [42], [44], recruits of red mullet and hake [45], and small-spotted catshark [46]. The shape of the structure or size of the patches varied between studies for several non-exclusive reasons, such as the scale of the sampling design, the spatial structure of forcing factors within each region, the bathymetry, the currents or the nature and distribution of the sediments. Nonetheless, the temporal stability of the variogram structures had, to our knowledge, never been explicitly tested. The spatial structures of demersal/benthic populations may occur at several scales because of biotic processes, such as intra-specific interactions, or a common response to an environmental forcing [47]. For instance, some species are known to depend on particular sediments, such as muddy seabed sediments for Norway lobster [48], sand and muddy bottoms for Mediterranean horse mackerel [49] muddy bottoms for red mullet [50] and coarse sediments for small-spotted catshark [46]. Using external factors within a spatial regression model could help in detecting the main processes that could cause such autocorrelation [51], [52]. More precisely, spatial autocorrelation would be modeled by introducing dependence in the residuals. If habitats variables are well identified, the spatial structure of the residuals would be attributed to the interaction of individuals.

Annual maps of species' log-densities were produced by kriging, using annual observations and the mean variogram. The mean variograms were clearly more robust because they were based on the whole dataset. Furthermore, their forms were smoother and the models fitted better. Note that the stability of the annual variograms is an indication of the stability of spatial structures but does not necessarily imply the stability of the spatial distributions. For instance, if the spatial structure is due to interactions among individuals, the population may move across the area through time (and thus change its spatial distribution), while keeping the same spatial structure. This was not the case here as the annual maps displayed similar patterns from year-to-year for all the species. EOF enabled us to detect for each species their most persistent patterns over time, which were different between species. As contributions from each year to the first EOF were relatively equal, part of the variance explained by the first EOF axis confirmed the persistence of the spatial distribution through the 17 years. The percentages of variance due to the first axis were globally high, about 71% on average and even more for the grey gurnard, Norway lobster and hake. Only the squids exhibited a relatively low percentage of variance (38%), possibly because this group includes three different species that may have dissimilar spatial distributions, implying additional variability. A recent study in the Tyrrhenian Sea [53] showed similar spatial patterns between years for the *Illex coindetii*, which tends to support such a hypothesis. Temporal stability of the spatial distributions is not so surprising for some species like the Norway lobster, due to its burrowing behavior and its dependence to particular sediments [42], [54]. However, such stability was not as expected for the other species, especially those more mobile, such as the hake, small-spotted catshark, and Atlantic and Mediterranean horse mackerels. These species are also known to perform ontogenetic migration [51], [52], which may generate variability in their spatial distribution. Our results are consistent with a few studies that have documented the temporal persistence of the nurseries of hake, red mullet and Norway lobster [9], [55].

The limit of the MEDITS sampling gear, which is more efficient for fishing on the shelf than at the upper slope where adults of several species are often concentrated, explains the relatively poor number of adults observed in some years for many species. This made a spatio-temporal analysis on only the adults intractable. Therefore, we produced average log-density maps in the 1994–2010 period, which was meaningful because of the temporal stability of annual spatial distributions. Adults were generally more concentrated than juveniles and occupied sub-areas within those of the juveniles. For instance, hake juveniles were homogeneously distributed in depths of 100 m to 200 m, whereas adults were more concentrated in depths of around 200 m. This is consistent with previous studies that describe an ontogenetic migration from the nurseries, located generally in shallow waters, to the end of the shelf, where spawning occurs [45], [55]–[59]. Our results on hake contradict a previous study that found more patchiness among younger hake individuals, ascribing it to their reduced active foraging abilities [60]. However, our results were averaged over 17 years, so that potential patchiness of juvenile distributions is smoothed. Red mullet nurseries were identified on the South Adriatic Coast in depths of up to 50 m, whereas we also found juveniles in depths of 100 m to 200 m, suggesting that their location is more dependent on the sediment type than the bathymetry. The main interest of our study on this specific issue is to show a common pattern across different species, but it is noticeable that the spatial distributions of adults *versus* those of juveniles remain poorly documented and need further investigations. From a fisheries-management viewpoint, the protection of nurseries could be insufficient if the adult populations are distributed elsewhere [61], [62]. Therefore, the occurrence of areas including both juveniles and adults is of peculiar interest. Such areas differed among species, but four zones can be identified: Mediterranean horse mackerel in shallow waters (10 m–50 m); Norway lobster on the slope; hake, Atlantic horse mackerel, angler and horned octopus at the end of the eastern side of the shelf (100 m–200 m), small-spotted catshark and grey gurnard on the opposite side, and red mullet on both sides (along the 100 m isobath). This relative complexity clearly illustrates the need to conduct a multivariate analysis to examine whether few strategic areas of interest could be identified within the Gulf of Lions.

The MEDITS survey is shown to be a suitable material for the study of the spatio-temporal patterns of marine species. Since 1994, it has been carried out in a standard manner (using the same sampling design, fishing procedure and gear). As we have already stressed, MEDITS bottom-trawls cannot, however, access the deepest areas of the continental slope because of the marine canyons. The biggest hake caught by Spanish longliners in this area are therefore not accessible to the MEDITS trawl survey. Furthermore, the survey occurs only once a year, for the month of June, so that year-to-year variations in abundance might partially reflect seasonal variations. While the MEDITS trawl survey obviously includes some limitations due to a restrictive sampling period, the high stability of the spatial distributions of all the species indicates that this survey is, nonetheless, able to capture the bulk of the spatial dynamics of these species. Another limitation in the ecological interpretation of our results was due to the identification uncertainty of some species, which forced us to mix several species of angler and squid. As these species are neither distinguished in the exploitation context, our results could, however, be of interest from a management viewpoint.

We also conducted the same analysis by transforming the densities into presence/absence data. This approach does not require data to be log-normally distributed and the binary transformation also removed the proportional effect. Simulation analysis was done by generating a binary field using the relation between a Gaussian and an indicator variogram [63]. The results were consistent with those from log-densities data, but the variograms were less structured. Furthermore, variograms of binary processes seem to be less robust and more sensitive than those obtained on log-densities. In general and counter-intuitively, the binary field seems to require a larger sample size for a good estimation of the variograms than with density data. The information on the presence of species is less informative than the log-density and the statistical conditions to infer spatial structures could be thus deteriorated. A compromise between these two approaches may be the indicator kriging [30], [64], which is also a free distribution method that takes into account the proportional effect by discretizing density values according to fixed thresholds [23]. Still, the choice of the number of thresholds may be a tricky question. However, from an ecological viewpoint it is important to note that all the above results (stability of the spatial structures and distributions) were robust to the data transformation.

Despite the strong persistence of the spatial distributions, we also detected a link between the expansion/contraction of the presence areas of these 12 key species and their log-abundance: the more abundant the species, the larger the presence area. The linear relationship was positive and significant for all the species, except for hake, Atlantic horse mackerel and gray gurnard, for which it was only positive. However, these species occupied almost all the study area, whatever their level of abundance. Therefore, our results tend to support MacCall's basin theory, which states that the presence area is positively correlated to the abundance. Geographical expansion at high levels of abundance were already observed among pelagic and demersal fish, such as haddock [65], [66], cod [67], [68], as well as hake, grey gurnard and angler [56], [69]. According to MacCall's theory, the spatial expansion of these species would result from density-dependent processes driven by habitat suitability, which depends on abiotic conditions, food limitations and predation avoidance. When density in most suitable habitats increases (e.g. because of higher recruitment success), the population may invade less suitable habitats, so that the expansion level depends on a trade-off between the density level inside the former habitat and the quality of the latter habitat. To investigate this issue more deeply, we also measured the log-abundance where species were systematically present over time (i.e. their optimal habitats). The positive and significant relationships between the log-abundance over the whole studied area and the log-abundance in their optimal habitats indicate that these species never saturated their main habitats. This suggests that these species were at abundance levels (far) below their carrying capacity. Such a result fits with the diagnosis of overexploitation of several of these species [70]. Note that the spatial expansion due to density-dependence processes assumed by the MacCall basin hypothesis does not necessary imply that the abundance in optimal habitat is saturated. Such a process can occur at different levels of abundance in the optimal habitats, so that abundance can increase in the optimal habitats during a period of spatial expansion [38], [71]. Alternatively or additionally, the spatial expansion/contraction of a population may also be driven by density-independent factors, like environmental changes (e.g. due to variations in the Rhone river run-off that is known to affect the sediments in the Gulf of Lions). Additional information about the habitats and the abiotic factors that affect species abundance is thus necessary to properly define the optimal habitats and to draw any conclusion.

Finally, our results give support to MPA as a pertinent management tool for exploited bentho-demersal species in the Gulf of Lions. Despite the high mobility of most of the species, their spatial distributions appear to be surprisingly stable over time. This was already empirically recognized for hake. Its relative resistance to overexploitation is supposed to be due to the persistence of large spawners at the end of the continental shelf and at the upper side of the slope, which are less accessible to fisheries [10]. This natural refuge has recently been recognized by the General Fisheries Commission for the Mediterranean (GFCM), which implemented a Fishery Restricted Area in the East side of the Gulf of Lions [70]. Note that areas where spawning grounds and nurseries overlap may also improve the performances of the MPAs [11]. Our study thus gives the first quantification of the spatial dynamics of hake and other overexploited species of the Gulf of Lions and could help in defining MPA in this area. If these species have the ability to diffuse outside their main habitats as abundance increases, MPAs could (partially) maintain fishery yields while enhancing their total abundance. However, this study is only a preliminary step and more work is needed. A deeper understanding about the density-dependent habitat selection would help to assess the potential benefits of MPAs for these populations. This would require the modeling of the habitat suitability of each species. This approach is not straightforward because the relationship between species abundance and external variables is difficult to assess in practice. A first exploratory analysis indicated that the relationships are complex, with non-linear shapes and interactions between the different factors. The implementation of a realistic model thus deserves deep investigation from a statistical viewpoint. Furthermore, an analysis that would take into account the fishing effort in surrounding areas should be done to fully quantify the performances of various designs of MPAs [72]. Another difficulty arises from the fact that these 12 species displayed different spatial distributions. As an example, the Fishery Restricted Area implemented on the eastern part of the slope essentially targets hake, angler, blue whiting, red shrimp and Norway lobster [9]. Therefore, a common set of MPAs to protect key exploited demersal species is rather challenging. A spatial and multivariate analysis including all targeted species or using biodiversity indicators will be necessary to detect such areas.

## Supporting Information

Figure S1 **Annual maps of anglers.** Kriged annual maps of both anglers (*Lophius budegassa* and *Lophius piscatorius*) log-density (Z) from 1994 to 2010.
(PNG)

Figure S2 **Annual maps of Atlantic horse mackerel.** Kriged annual maps of Atlantic horse mackerel (*Trachurus trachurus*) log-density (Z) from 1994 to 2010.
(PNG)

Figure S3 **Annual maps of Mediterranean horse mackerel.** Kriged annual maps of Mediterranean horse mackerel (*Trachurus mediterraneus*) log-density (Z) from 1994 to 2010.
(PNG)

Figure S4 **Annual maps of grey gurnard.** Kriged annual maps of grey gurnard (*Eutrigla gurnardus*) log-density (Z) from 1994 to 2010.
(PNG)

Figure S5 **Annual maps of Norway lobster.** Kriged annual maps of Norway lobster (*Nephrops norvegicus*) log-density (Z) from 1994 to 2010.
(PNG)

Figure S6 **Annual maps of red mullet.** Kriged annual maps of red mullet (*Mullus barbatus*) log-density (Z) from 1994 to 2010.
(PNG)

Figure S7 **Annual maps of horned octopus.** Kriged annual maps of horned octopus (*Eledone cirrhosa*) log-density (Z) from 1994 to 2010.
(PNG)

Figure S8 **Annual maps of small-spotted catshark.** Kriged annual maps of small-spotted catshark (*Scyliorhinus canicula*) log-density (Z) from 1994 to 2010.
(PNG)

Figure S9 **Annual maps of cuttlefish.** Kriged annual maps of cuttlefish (*Sepia Elegans*) log-density (Z) from 1994 to 2010.
(PNG)

Figure S10 **Annual maps of squids.** Kriged annual maps of squids (*Illex coindetti*, *Todarodes sagittatus* and *Todaropsis eblanae*) log-density (Z) from 1994 to 2010.
(PNG)

Figure S11 **Average distributions of juveniles** ***versus*** **adults.** Average maps (1994–2010) of the log-density (Z) of juveniles and adults of *Trachurus trachurus*, *Trachurus Mediterraneus*, *Eutrigla gurnardus*, *Nephrops norvegicus*, *Mullus barbatus* and *Eledone cirrhosa*.
(PNG)

Figure S12 **Correlation circles from the EOF analysis.** The correlation circle depicts the contribution of each descriptor (i.e. the years) to the two first axes of the Empirical Orthogonal Functions (EOF). Higher the contribution of a given descriptor to the first EOF axis, longer the arrow. Here, all the descriptors have positive and high contributions to the first axis, which insured that the percentage of variance explained by this EOF is an indicator of the stability of the spatial distributions through time.
(PDF)

Figure S13 **Gravity centers and inertia.** For each key species, gravity centers (crosses) and inertia axes of annual log-density maps, from 1994 to 2010.
(TIF)
