# Supplementary figures and images for: Spatio-Temporal Patterns of Key Exploited Marine Species in the Northwestern Mediterranean Sea
Source: PLoS One. 2012 May 24;7(5):e37907. doi: 10.1371/journal.pone.0037907 (PMC3360014; doi:10.1371/journal.pone.0037907)

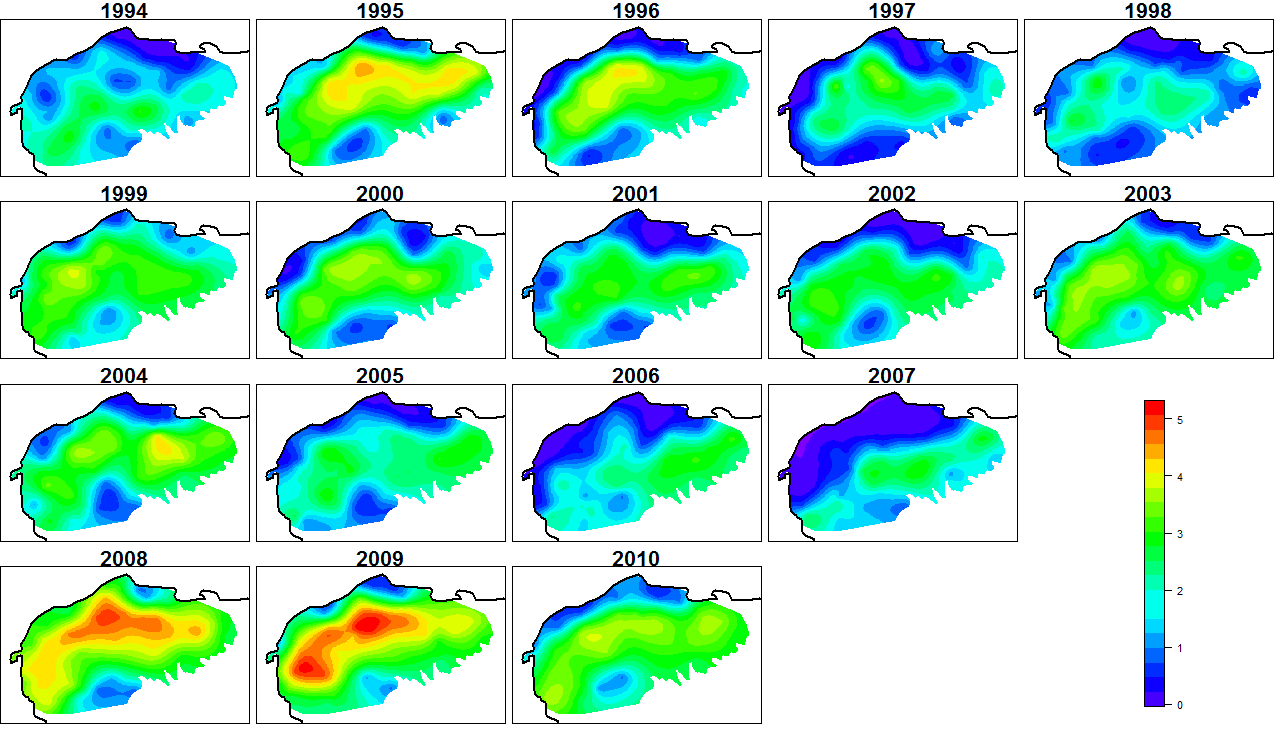

Supplement: Figure S1 — Annual maps of anglers. Kriged annual maps of both anglers (Lophius budegassa and Lophius piscatorius) log-density (Z) from 1994 to 2010. (PNG) [file pone.0037907.s001.png]

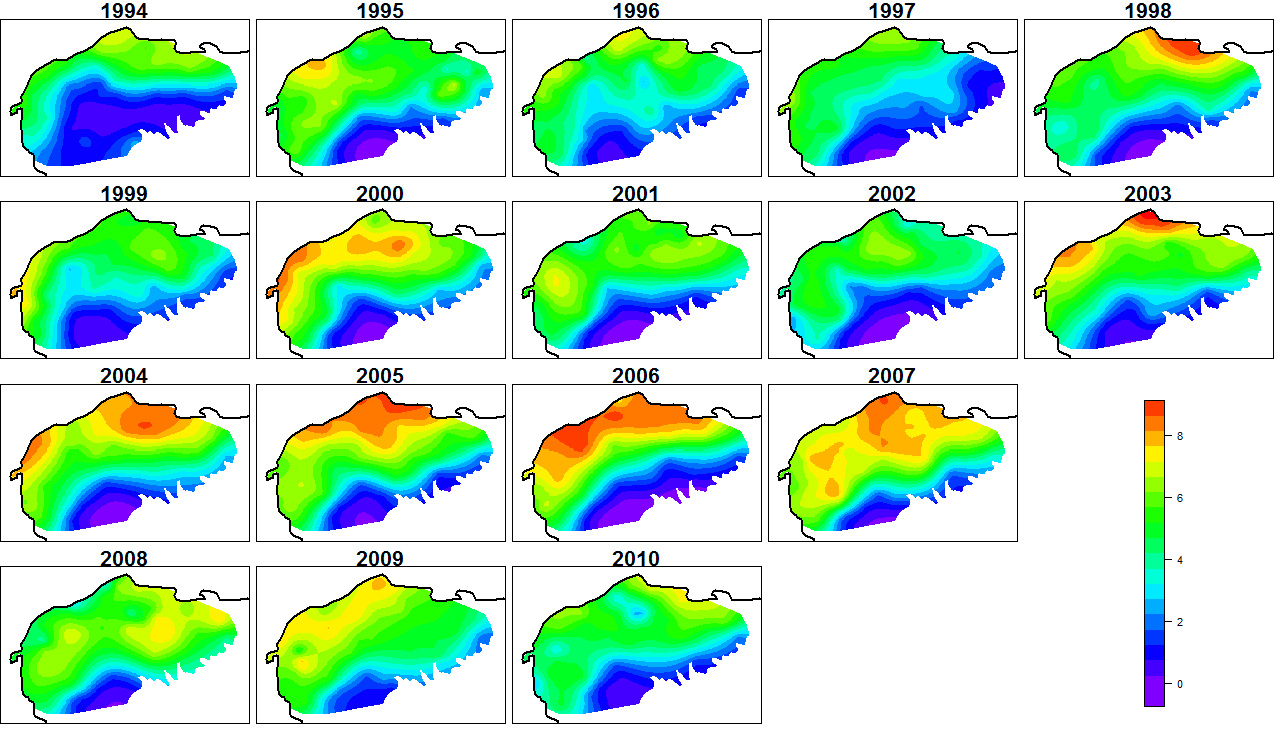

Supplement: Figure S2 — Annual maps of Atlantic horse mackerel. Kriged annual maps of Atlantic horse mackerel (Trachurus trachurus) log-density (Z) from 1994 to 2010. (PNG) [file pone.0037907.s002.png]

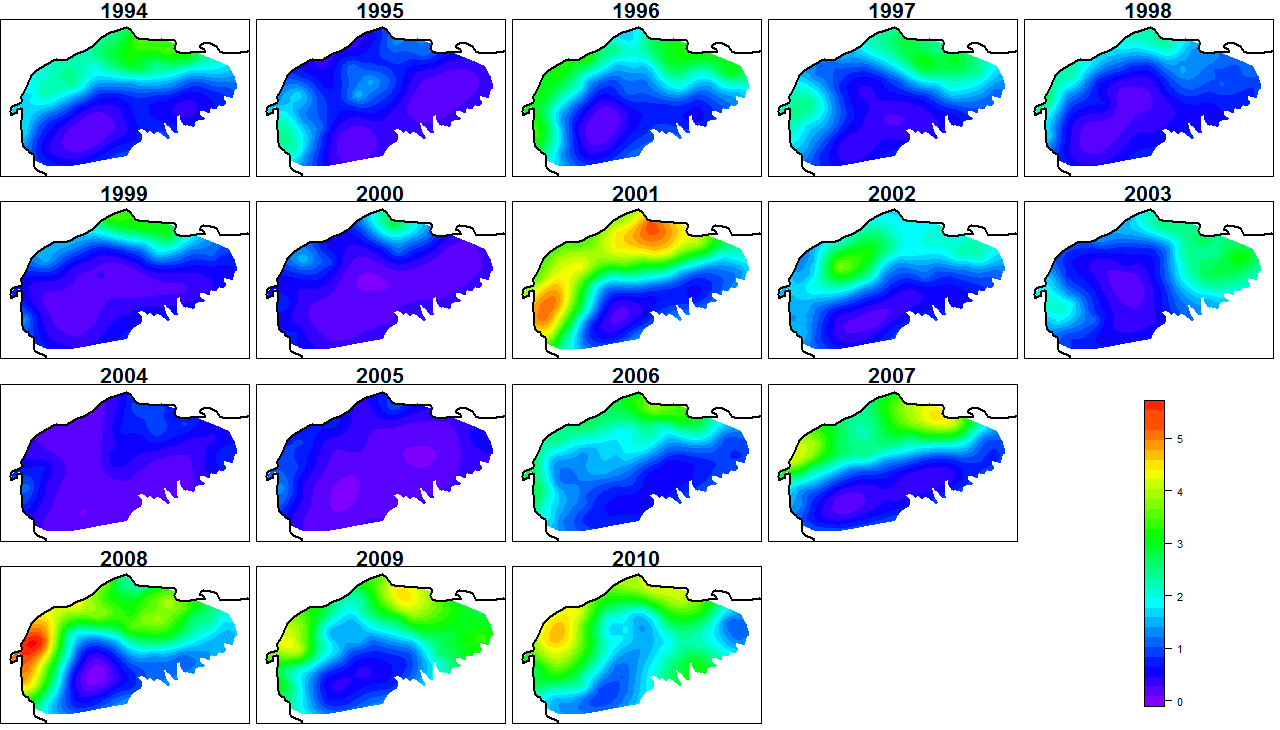

Supplement: Figure S3 — Annual maps of Mediterranean horse mackerel. Kriged annual maps of Mediterranean horse mackerel (Trachurus mediterraneus) log-density (Z) from 1994 to 2010. (PNG) [file pone.0037907.s003.png]

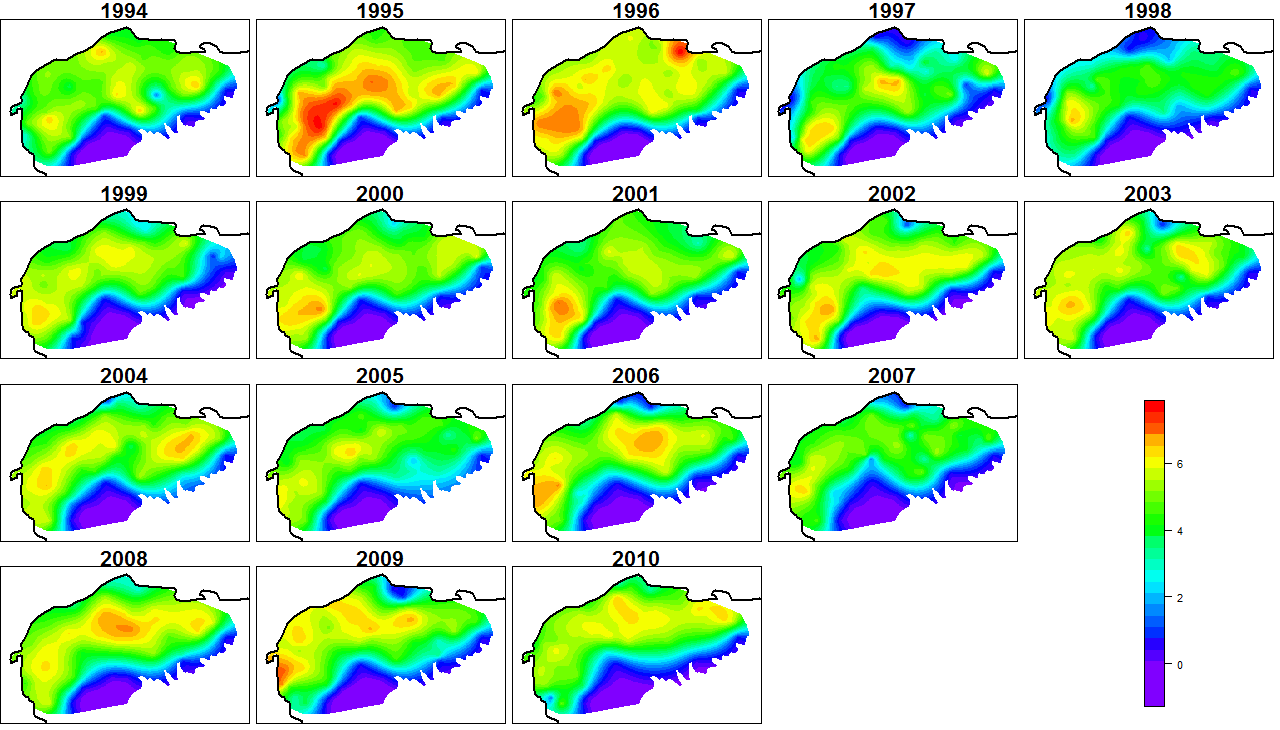

Supplement: Figure S4 — Annual maps of grey gurnard. Kriged annual maps of grey gurnard (Eutrigla gurnardus) log-density (Z) from 1994 to 2010. (PNG) [file pone.0037907.s004.png]

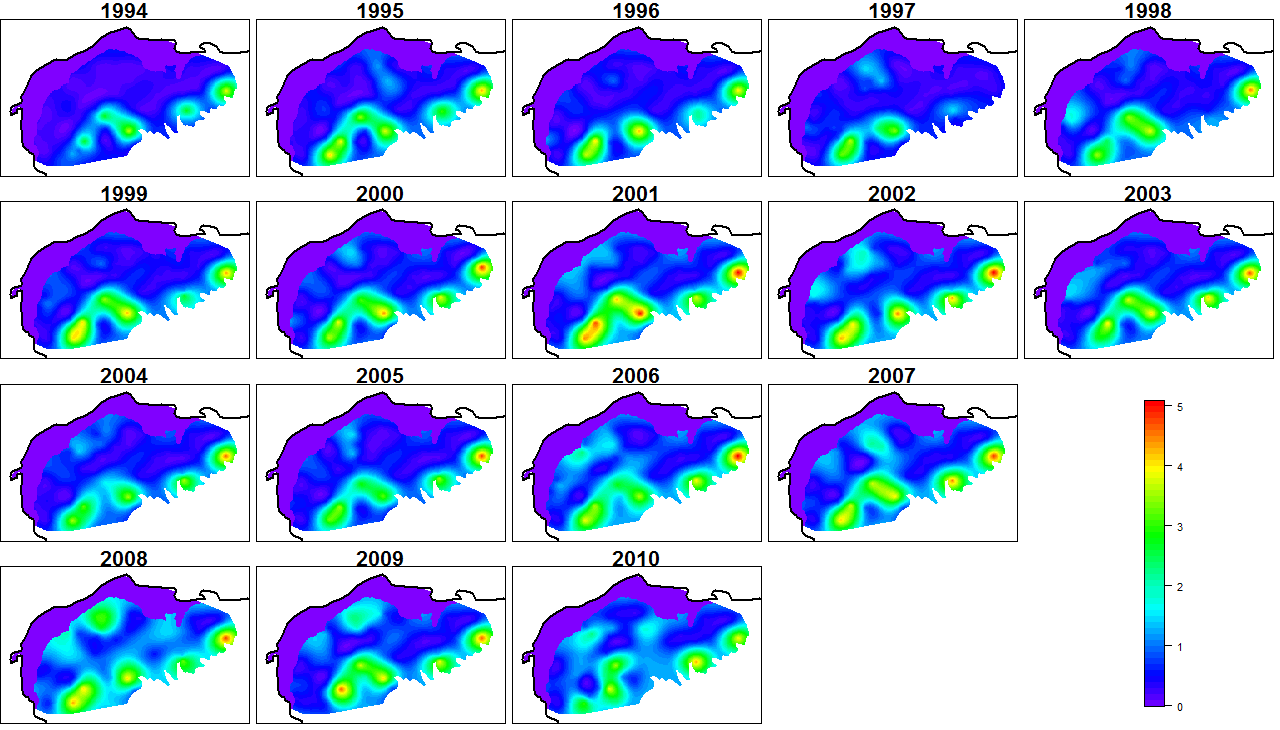

Supplement: Figure S5 — Annual maps of Norway lobster. Kriged annual maps of Norway lobster (Nephrops norvegicus) log-density (Z) from 1994 to 2010. (PNG) [file pone.0037907.s005.png]

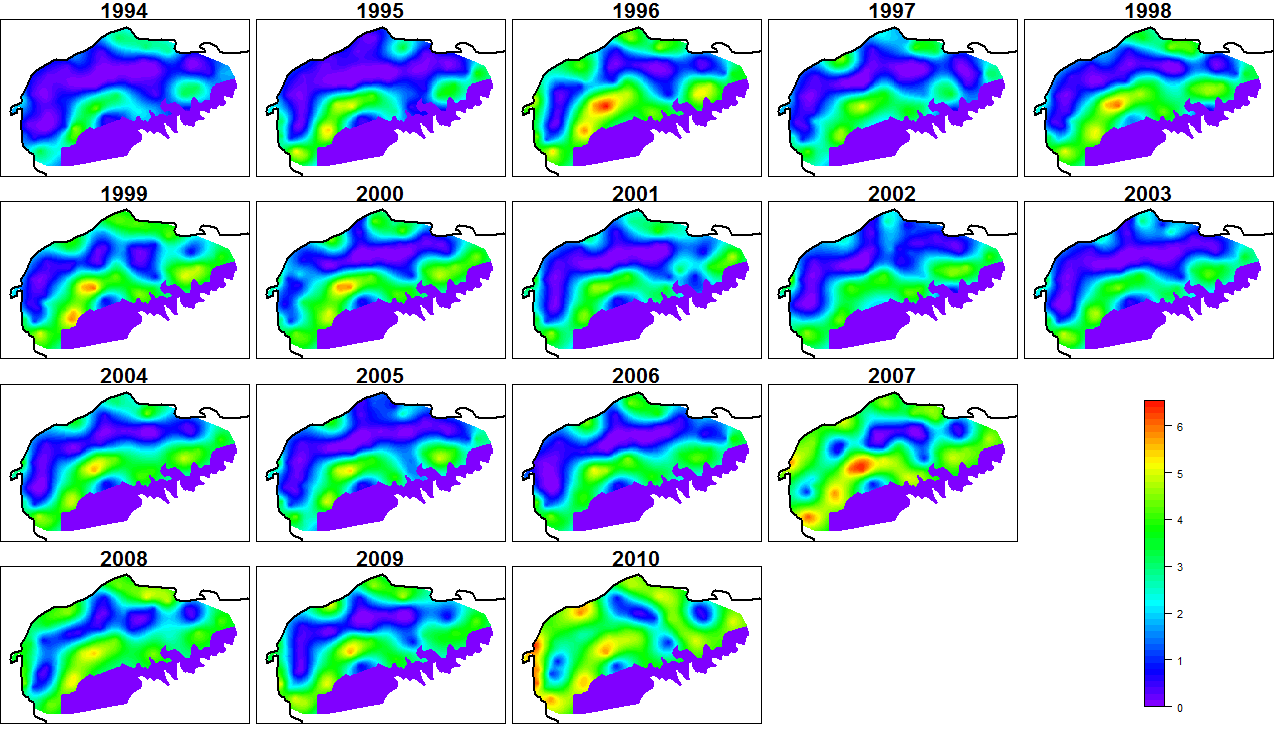

Supplement: Figure S6 — Annual maps of red mullet. Kriged annual maps of red mullet (Mullus barbatus) log-density (Z) from 1994 to 2010. (PNG) [file pone.0037907.s006.png]

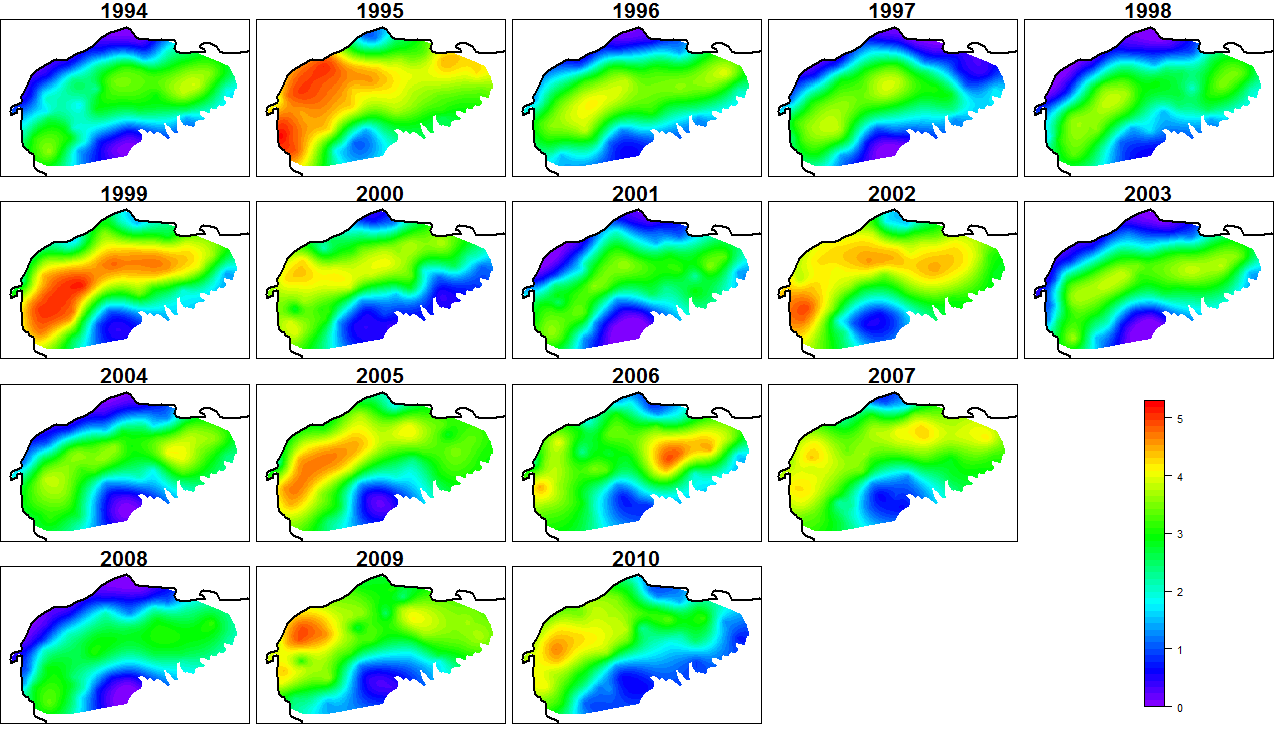

Supplement: Figure S7 — Annual maps of horned octopus. Kriged annual maps of horned octopus (Eledone cirrhosa) log-density (Z) from 1994 to 2010. (PNG) [file pone.0037907.s007.png]

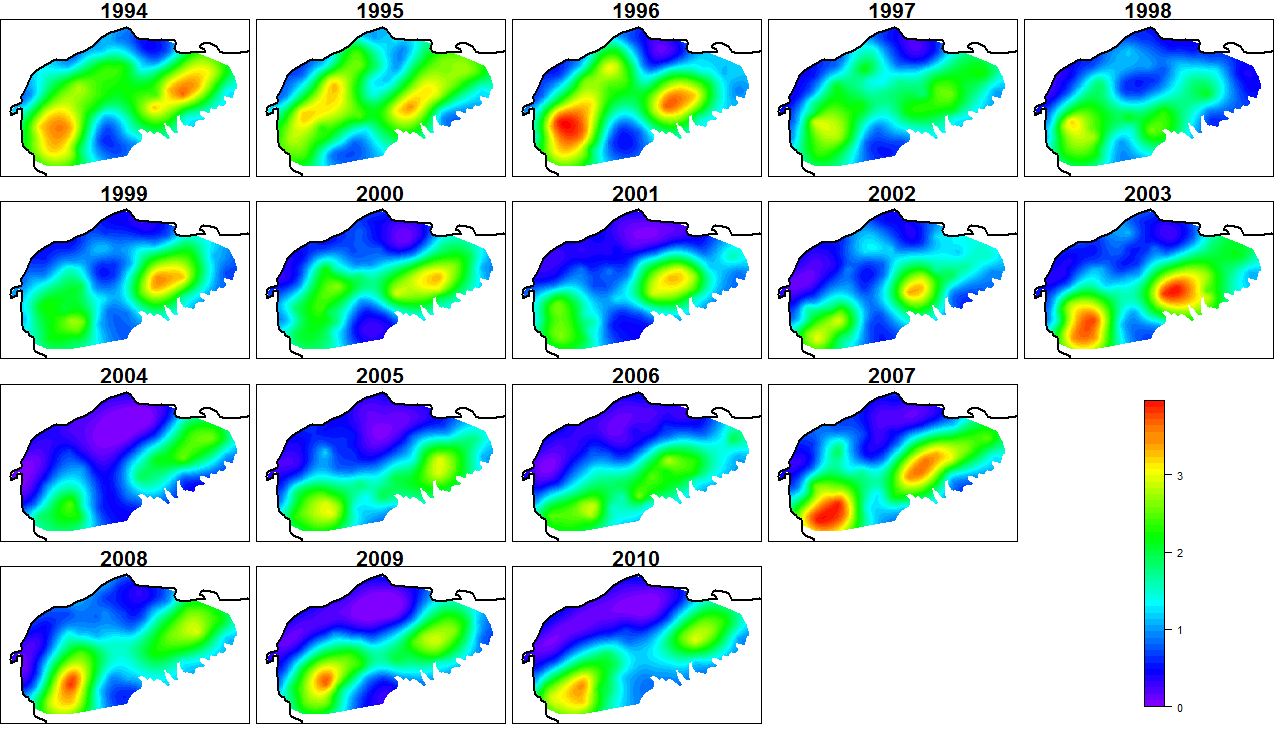

Supplement: Figure S8 — Annual maps of small-spotted catshark. Kriged annual maps of small-spotted catshark (Scyliorhinus canicula) log-density (Z) from 1994 to 2010. (PNG) [file pone.0037907.s008.png]

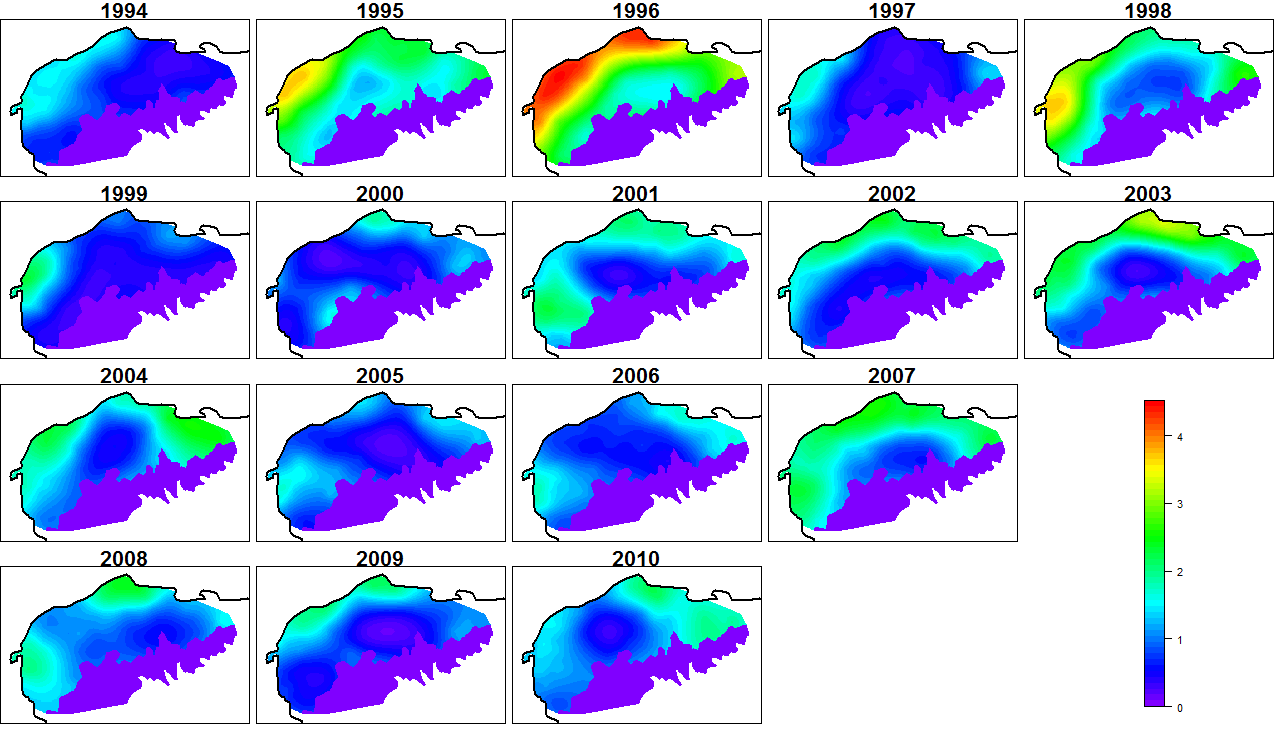

Supplement: Figure S9 — Annual maps of cuttlefish. Kriged annual maps of cuttlefish (Sepia Elegans) log-density (Z) from 1994 to 2010. (PNG) [file pone.0037907.s009.png]

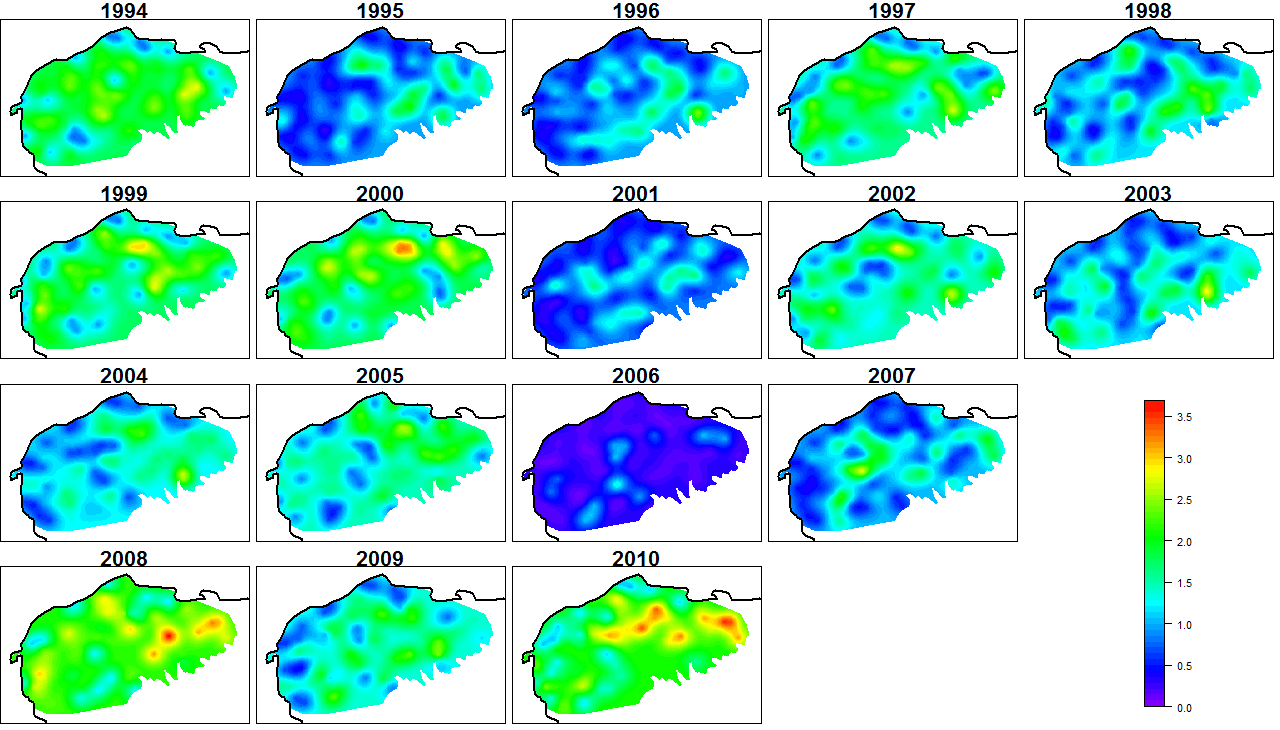

Supplement: Figure S10 — Annual maps of squids. Kriged annual maps of squids (Illex coindetti, Todarodes sagittatus and Todaropsis eblanae) log-density (Z) from 1994 to 2010. (PNG) [file pone.0037907.s010.png]

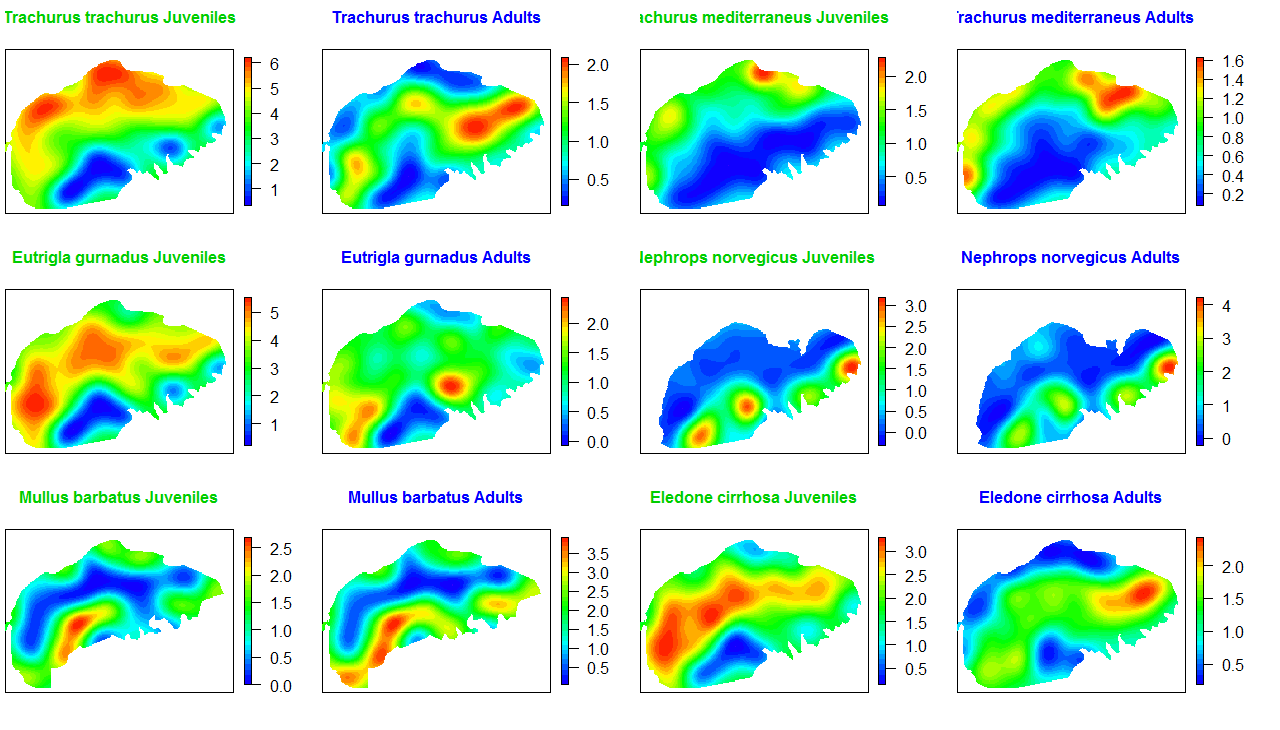

Supplement: Figure S11 — Average distributions of juveniles versus adults. Average maps (1994–2010) of the log-density (Z) of juveniles and adults of Trachurus trachurus, Trachurus Mediterraneus, Eutrigla gurnardus, Nephrops norvegicus, Mullus barbatus and Eledone cirrhosa. (PNG) [file pone.0037907.s011.png]

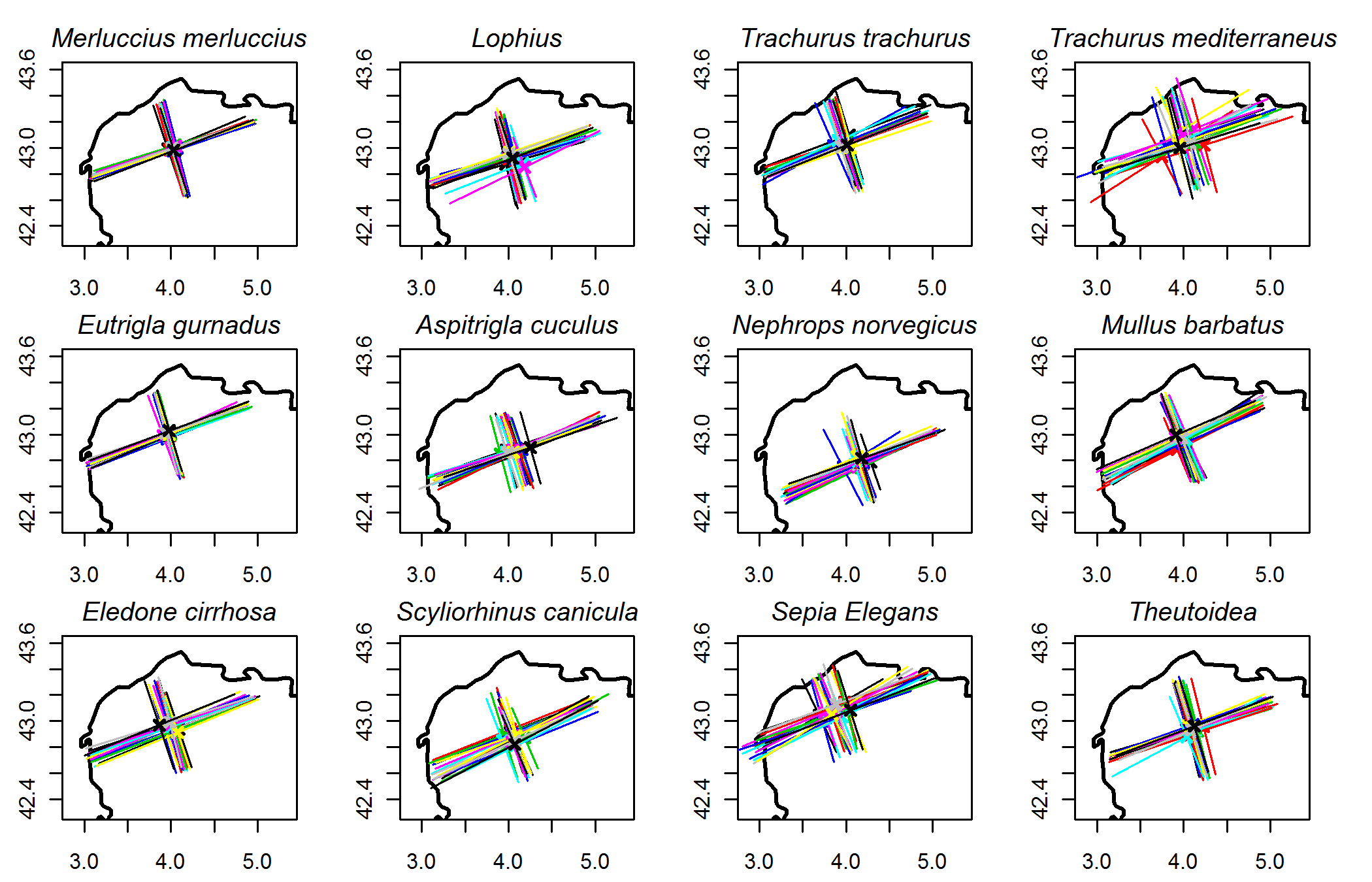

Supplement: Figure S13 — Gravity centers and inertia. For each key species, gravity centers (crosses) and inertia axes of annual log-density maps, from 1994 to 2010. (TIF) [file pone.0037907.s013.tif]
