# Supplementary material for: Spatio-Temporal Patterns of Key Exploited Marine Species in the Northwestern Mediterranean Sea
Source: PLoS One. 2012 May 24;7(5):e37907. doi: 10.1371/journal.pone.0037907 (PMC3360014; doi:10.1371/journal.pone.0037907)
Supplement: Figure S12 — Correlation circles from the EOF analysis. The correlation circle depicts the contribution of each descriptor (i.e. the years) to the two first axes of the Empirical Orthogonal Functions (EOF). Higher the contribution of a given descriptor to the first EOF axis, longer the arrow. Here, all the descriptors have positive and high contributions to the first axis, which insured that the percentage of variance explained by this EOF is an indicator of the stability of the spatial distributions through time. (PDF) [file pone.0037907.s012.pdf]

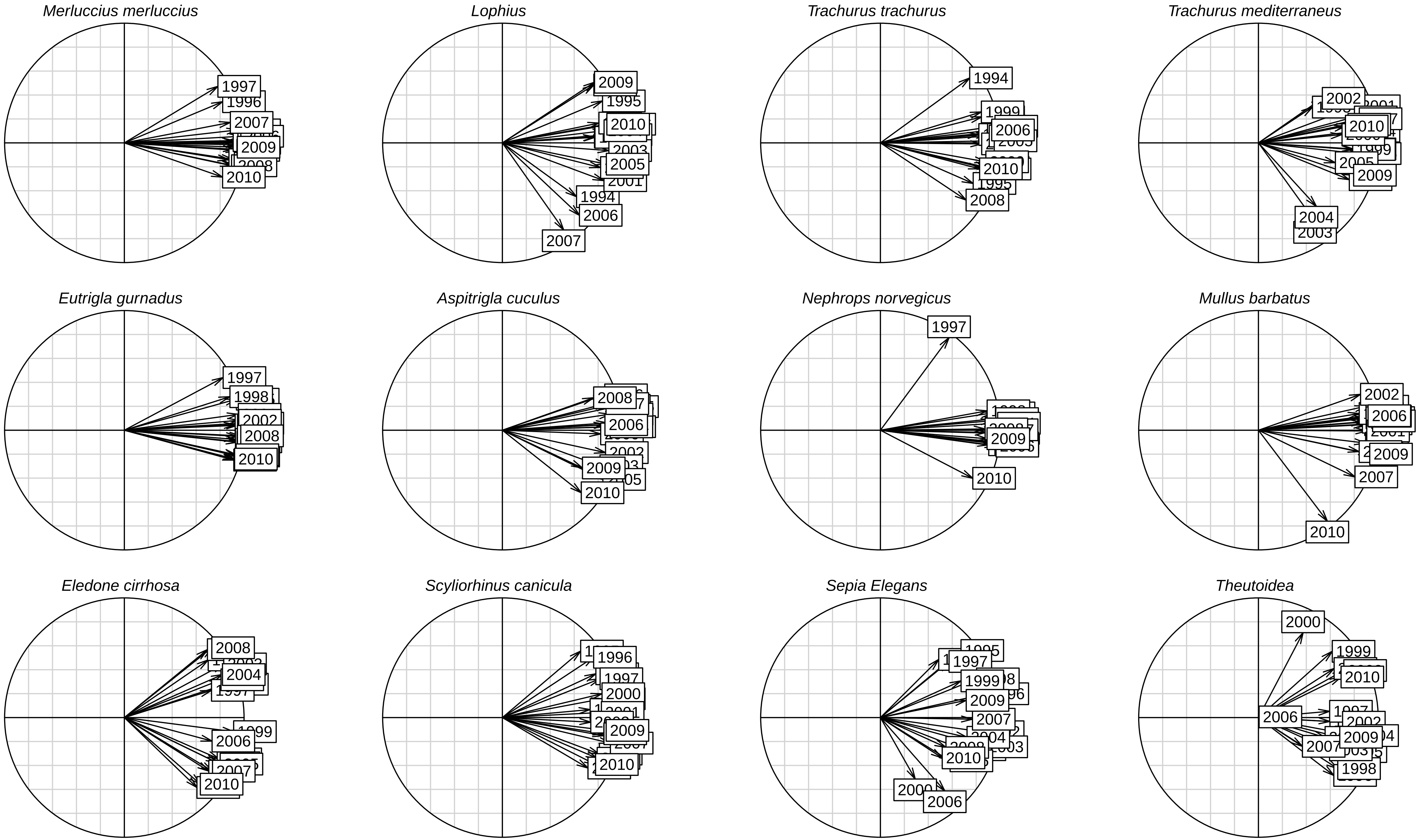
Merluccius merluccius
1997
1996
2007
2009
2010
Lophius
2009
1995
2010
2003
2005
1994
2006
2007
Trachurus trachurus
1994
1999
2006
2010
2008
Trachurus mediterraneus
2002
2010
2005
2009
2004
2003
Eutrigla gurnadus
1997
2002
2008
2010
Aspitrigla cuculus
2008
2006
2002
2009
2010
Nephrops norvegicus
1997
2009
2010
Mullus barbatus
2002
2006
2009
2007
2010
Eledone cirrhosa
2008
2004
1999
2006
2007
2010
Scyliorhinus canicula
1996
1997
2000
2009
2010
Sepia Elegans
1997
1999
2009
2007
2010
2000
2006
Theutoidea
2000
1999
2010
2006
2002
2009
2007
1998
